# Serum regenerative islet-derived protein 1α: a novel and sensitive biomarker for endoscopic disease activity in ulcerative colitis

**DOI:** 10.1080/07853890.2025.2496404

**Published:** 2025-04-28

**Authors:** Xiaoduan Zhuang, Huiyue Jiang, Tingting Fan, Xiaoqi Luo, Xuanfang Zhong, Jian Guo, Yaxian Zhou, Bingsheng Li, Xinying Wang

**Affiliations:** ^a^ Department of Gastroenterology, Zhujiang Hospital, Southern Medical University, Guangzhou, China; ^b^ Department of Gastroenterology, The People’s Hospital of Baoan Shenzhen, Shenzhen, China; ^c^ Department of Gastroenterology, Huizhou First Hospital, Huizhou, China; ^d^ Senboll Biotechnology Co., Ltd, Pingshan Bio-Pharmacy Business Accelerator, Shenzhen, Guangdong, China

**Keywords:** Mucosal healing, ulcerative colitis, regenerative islet-derived protein 1α, C-reactive protein

## Abstract

**Introduction:**

Mucosal healing (MH) has established as a long-term therapeutic goal for inflammatory bowel disease (IBD). Regenerative Islet-derived Protein 1α (reg1α) was reported to be closely related to gastrointestinal mucosal injury; however, its potential in monitoring MH remains unexplored.

**Methods:**

Serum reg1α levels were quantified in 156 consecutive IBD patients (January 2021-December 2023) and stratified by endoscopic findings into MH (*n* = 136) and non-mucosal healing (NMH) groups. Diagnostic performance of reg1α was evaluated and compared to C-reactive protein (CRP) using receiver operating characteristic analysis.

**Results:**

A total of 136 patients (85 with CD and 51 with UC) were finally included. Serum reg1α levels were significantly correlated with endoscopic activity. Patients in NMH group demonstrating markedly higher reg1α levels than those in MH group (92.6 *vs*. 55.5 ng/ml, *p* < 0.001). In UC, reg1α outperformed CRP in sensitivity (82.4% *vs*. 55.9%, *p* = 0.012) and accuracy (80.4% *vs*. 70.6%, *p* = 0.001) for monitoring MH. This superiority persisted in UC subgroups with non-or mild clinical symptoms. Combined index (reg1α + CRP) and multivariate model (incorporating clinical indices, reg1α and CRP) also enhanced sensitivity and accuracy over CRP alone in UC (all *p* < 0.05). However, reg1α showed no significantly higher efficacy in monitoring NMH compared to CRP in CD. In CRP-normal patients, patients with NMH had higher reg1α levels than those with MH in UC (77.6 *vs*. 49.8 ng/ml, *p* = 0.018), but not in CD.

**Conclusion:**

Reg1α represents as a novel and sensitive biomarker of endoscopic disease activity in patients with UC, even in patients with non- or mild clinical symptoms or normal CRP levels.

## Introduction

The incidence of inflammatory bowel diseases (IBD), including Crohn’s disease and ulcerative colitis, is increasing globally [1,2]. Owing to uncontrolled intestinal inflammation, patients with IBD may develop several complications, such as colon strictures and gut perforations [3,4]. Consequently, discovering effective treatment strategies to achieve gut mucosal healing (MH) has been a long-term therapeutic goal for IBD over the past 20 years [5,6]. Several studies have reported that MH significantly ameliorates complications and the need for surgery [7–9]. Endoscopy is considered the gold standard for evaluating MH in patients with IBD; however, frequent routine examinations are not optimal in clinical practice because of their invasiveness, high cost, discomfort, and impracticality in patients with vital organ dysfunction [10].

Non-invasive and reproducible serum biomarkers, such as C-reactive protein (CRP) [11], leucine-rich alpha-2 glycoprotein [12,13], and plasminogen activator inhibitor 1 [14] have been developed and recommended for monitoring MH. However, the above biomarkers are not specific to IBD. Recently, CRP, a validated biomarker for IBD, correlated unsatisfactorily with endoscopic activity in patients with IBD [15]. Especially in UC phenotype, the efficacy of CRP for mucosal healing is more insufficiency [12]. CRP synthesis in the liver is induced by circulating IL-6; however, UC is characterized by superficial inflammation limited to the mucosa, which may not show systemic response related to circulating IL-6 [16]. Moreover, inflammation occasionally accompanies endoscopy in patients with normal CRP levels [15]. Therefore, a reliable surrogate blood-based biomarker with higher sensitivity for MH is necessary, especially for UC phenotype. Human regenerative islet-derived protein 1α (reg1α) is a 16-kDa acidic phosphorylated glycoprotein encoded by the *REG1A* gene [17], which is expressed by pancreatic acinar cells, enterochromaffin-like cells, and all types of gastrointestinal epithelial cells. As a novel proliferative factor, reg1α plays an important role in ameliorating gastrointestinal mucosal injury. *REG1A* overexpression significantly alleviates dextran sodium sulfate-induced colitis and maintains the integrity of the mucosal barrier [18]. In patients with active CD, *REG1A* was significantly up-regulated [19]. In patients with UC, gene microarray and immunohistochemistry revealed that reg1α levels in those with clinical and histological inflammation was significantly higher than in those with clinical and histological remission [20]. Unlike CRP’s exclusive IL-6 dependence, it has been reported that the synthesis of reg1α in the digestive system can be not only induced by IL-6 but also by IL-22 and IL-8 [18,21,22]. This polyfunctional regulation mechanism may enable reg1α as a superior biomarker candidate for monitoring mucosal healing, offering enhanced sensitivity as compared with CRP.

Although reg1α is a promising biomarker for monitoring MH in IBD treatment, its monitoring value and clinical utility in real-world populations remains uncertain. Therefore, we aimed to explore the relationship between the serum reg1α level and endoscopic and clinical activity in IBD and compare the monitoring efficacy of reg1α for MH with that of CRP, especially in UC phenotype.

## Materials and methods

### Patients

One hundred and fifty-five consecutive patients with IBD who underwent both endoscopy and serum reg1α testing at Zhujiang Hospital, Southern Medical University (Guangzhou, China) (*n* = 132) and the first people’s hospital of Huizhou City (Huizhou, China) (*n* = 23) between January 2021 and December 2023 were enrolled. Among the 155 patients, 19 were excluded due to the following reasons: (i) the interval between serum reg1α testing and endoscopy exceeded 7 days (*n* = 5), (ii) excessive outliers (*n* = 2), (iii) incomplete medical records (*n* = 5), (iv) incomplete gastrointestinal endoscopic examination (*n* = 5), (v) patients with cancer and infection (*n* = 2). Overall, 136 patients (UC, 51; CD, 85) participated in this study. This study was conducted in accordance with the Declaration of Helsinki and was approved by the Ethics Committee of Zhujiang Hospital (approval number: 2021-KY-115-03). Written informed consent was obtained from all the study participants.

### Assessment of clinical and endoscopic activities in IBD

Clinical activity (CA) was assessed by the physician in charge using the Harvey–Bradshaw index (HBI) for patients with CD and the partial Mayo clinical score (pMayo) for patients with UC. Clinical remission (CR) was defined as an HBI of <5 for CD and an pMayo of 0–1 for UC. Mild, moderate, and severe CAs were defined as HBI of 5–7, 8–16, and ≥17 in patients with CD [23] and pMayo of 2–4, 5–7, and ≥8 in patients with UC, respectively [24].

Colonoscopy and/or balloon-assisted small bowel endoscopy (BAE) were performed depending on the location of the lesions. Patients with lesions confined to the colon or terminal ileum underwent colonoscopy, whereas those with lesions in the small bowel beyond the terminal ileum underwent BAE. Endoscopic activity was determined using the Simple Endoscopic Scale for Crohn’s Disease (SES-CD) for patients with CD and the Mayo Endoscopic Score (MES) for patients with UC. The SES-CD assesses four parameters (ulcer size, ulcerated surface area, extent of lesions, and stenosis, each scored from 0 to 3) across four colonic segments (terminal ileum, right hemi-colon, transverse colon, left hemi-colon, and rectum) [25]. The total score derived from these segments was utilized for analysis. For the MES evaluation, each segment of the colorectum (cecum and ascending colon combined, transverse colon, descending colon, sigmoid colon, and rectum) was examined, and the highest score observed in any segment of the colorectum for each patient was used for analysis [26]. MH was defined as an SES-CD of 0–2 for CD and an MES of 0–1 for UC; Non-mucosal healing (NMH) was defined as an SES-CD of ≥3 or the presence of ulcerations in CD and an MES of ≥2 for UC [5,26]. Mild, moderate, and severe endoscopic activity in CD were defined as SES-CD of 3–6, 7–15, and ≥16, respectively [27]. Moderate or severe endoscopic activity in UC was defined as an MES of ≥2. Endoscopic activity was independently scored by two endoscopists, and the scores were reviewed to see if they were different. Physicians who scored the clinical or endoscopic activity were blinded to the serum biomarker results.

### Measurement of serum reg1α levels

Blood samples were collected in the morning from the participants in a nonfasted state. The serum samples were collected by centrifuging the blood samples at 1500 rpm for 15 min, dividing them into aliquots, and storing them at −80 °C until use. Serum reg1α levels were measured using a qualitative enzyme-linked immunosorbent assay kit (Inova Diagnostics, USA) per the manufacturer’s instructions.

### Determination of CRP and other laboratory indicator levels

The levels of laboratory-measured indicators, including CRP and albumin, were measured. The blood samples for serum CRP, reg1α level tests and routine blood test were collected on the same day. Routine blood tests to determine the level of white blood cells, platelets, and erythrocyte sedimentation rate (ESR) were performed using the 5-Part Differential Hematology analyzer BC-6800PLUS (Mindray, Shenzhen, China). According to routine laboratory reference values, a normal CRP level was defined as a CRP level of <3 mg/L.

### Statistical analysis

The Chi-square, *t*, and Mann–Whitney U-tests (for data with non-normal distribution) were used for comparison between groups. The correlation between serum reg1α levels and endoscopic activity was assessed using Spearman’s rank correlation analysis. Longitudinal changes in serum reg1α levels among IBD patients were analysed *via* paired-sample *t*-test. A logistic regression model incorporating reg1α, CRP and clinical indices was developed to monitor NMH. Diagnostic performance of reg1α, CRP, combining index (reg1α + CRP), and model (reg1α, CRP and clinical indices) for NMH was evaluated through receiver operating characteristic (ROC) curve analysis, with DeLong’s test comparing area under the curve (AUC) values. The area under the curves (AUCs) were compared using DeLong’s test. Optimal diagnostic thresholds determined by Youden’s index enabled calculation of sensitivity, specificity, accuracy, positive likelihood ratio (PLR), and negative likelihood ratio (NLR). McNemar’s chi-square test was employed to compare the sensitivity, specificity, and accuracy.

All statistical analyses were performed using SPSS software (version 27.0; IBM Corp., Armonk, NY, USA). Statistical significance was defined as *p* < 0 .05.

## Results

### Patients’ clinical characteristics

**Figure 1.**
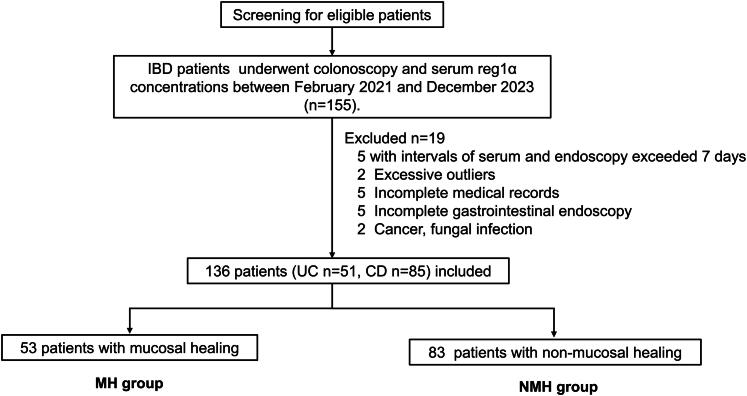
Patient inclusion flowchart. Abbreviations: IBD, Inflammatory bowel disease; CD, Crohn’s disease; UC, Ulcerative colitis; MH, Mucosal healing; NMH, Non-mucosal healing.

Overall, 136 patients (85 with CD and 51 with UC) with a median age of 36.7 ± 15.3 years (range: 14–72 years) were finally included in the study (Figure 1). Based on endoscopic findings, 53 (39.0%) and 83 (61.0%) patients were assigned to the MH and NMH groups, respectively. Clinical characteristics, including age, sex ratio, body mass index, smoking habits, diabetes, and IBD phenotype, were not significantly different between the two groups (all *p* > 0.05, Table 1). MES and SES-CD scores were significantly higher in the NMH group than in the MH group (2.4 ± 0.5 *vs*. 0.5 ± 0.5, *p* < 0.001; 10.4 ± 9.0 *vs*. 1.0 ± 0.9, *p* < 0.001, respectively). Regarding serum biomarkers, serum reg1α and CRP levels were significantly higher in the NMH group than in the MH group [92.6 (66.5, 126.8) *vs*. 55.5 (38.0, 82.5), *p* < 0.001; 6.8 (1.1, 23.6) *vs*. 0.5 (0.5, 2.1), *p* < 0.001, respectively].

**Table 1.** Clinical characteristics of the patients.

Variable	NMH (*n* = 83)	MH (*n* = 53)	*P*
Age, year, mean ± SD	37.1 ± 16.9	36.1 ± 12.4	0.695
Female/Male	26/57	16/37	0.889
BMI, mean ± SD	20.6 ± 4.0	21.2 ± 3.9	0.365
Smoker, *n*%	3 (3.7%)	3 (5.7%)	0.679
Diabetes, *n*%	2 (2.4%)	1 (1.9%)	1.000
Disease duration, year, median (IQR)	1 (0, 3)	3 (1, 7)	**0.007**
IBD phenotype, *n*%			0.296
UC	34 (41.0%)	17 (32.1%)	
CD	49 (59.0%)	36 (67.9%)	
MES in UC, mean ± SD	2.4 ± 0.5	0.5 ± 0.5	**<0.001**
SES-CD in CD, mean ± SD	10.4 ± 9.0	1.0 ± 0.9	**<0.001**
**Laboratory data**			
reg1α, ng/ml, median (IQR)	92.6 (66.5,126.8)	55.5 (38.0,82.5)	**<0.001**
CRP, mg/L, median (IQR)	6.8 (1.1,23.6)	0.5 (0.5,2.1)	**<0.001**
Alb, g/dL, median (IQR)	38.3 (32.6,41.6)	43.4 (40.8,46.7)	**<0.001**
Hb, g/dL, median (IQR)	120 (103,135)	133 (120,145)	**0.001**
ESR, mm/h, median (IQR)	34 (19,69)	12 (6,31)	**<0.001**
WBC*10^9^/mL, median (IQR)	7.1 (5.4,9.6)	6.2 (5.4,7.7)	**0.076**
PLT*10^6^/mL, median (IQR)	338 (235,415)	251 (218,293)	**0.001**

Abbreviations: NMH, Non-mucosal healing; MH, Mucosal healing; IBD, Inflammatory bowel disease; CD, Crohn’s disease; UC, Ulcerative colitis; MES, Mayo endoscopic score; SES-CD, Simple Endoscopic Score for Crohn’s disease; reg1α, regenerating islet-derived 1 alpha; CRP, C-reaction protein; Alb: Albumin; Hb, Haemoglobin; ESR, Erythrocyte sedimentation rate; WBC, White blood cell; PLT, Platelet; BMI, Body Mass Index; IQR, Interquartile range; SD, Standard deviation; P, P value.

### Correlations between serum reg1α level and endoscopic activity

**Figure 2.**
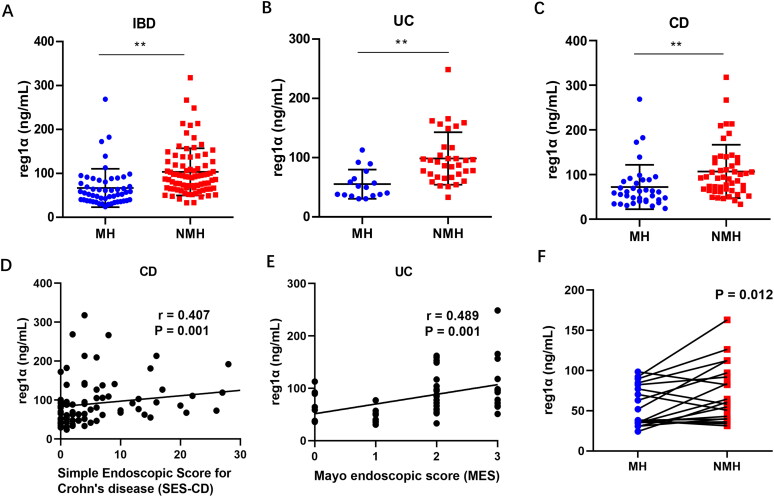
Correlation between reg1α and mucosal healing. Serum Reg1α levels between MH and NMH groups in IBD patients (a), CD phenotype (B) and UC phenotype (C); correlation of the MES (D) and SES-CD (E) scores with serum reg1α levels; paired analysis of IBD patients with NMH revealed a significantly decreased serum reg1α levels after achieving MH (10 pairs, F). *p < 0.05, **p < 0.001. Abbreviations: MES, Endoscopic Mayo score; SES-CD, Simple Endoscopic Score for Crohn’s disease; r, Correlation coefficient; reg1α, regenerating islet-derived 1 alpha; IBD, Inflammatory bowel disease; CD, Crohn’s disease; UC, Ulcerative colitis; MH, Mucosal healing; NMH, Non-mucosal healing.

First, we compared the serum reg1α levels in patients with IBD between the MH and NMH groups. Significantly higher reg1α levels were detected in patients in the NMH group than in patients in the MH group [92.6 (66.5, 126.8) *vs*. 55.5 (38.0, 82.5), *p* < 0.001, Figure 2(A)]. Similar trends were observed in the subgroups of patients with CD and UC [92.6 (64.6–131.9) *vs*. 60.0 (41.2–87.4), *p* < 0.001; 89.9 (67.0–117.1) *vs*. 49.8 (36.5–70.7), *p* < 0.001, respectively, Figure 2(B–C)]. Second, correlations between serum reg1α levels and disease-related endoscopic activity were examined in patients with CD and UC. Serum reg1α levels have significant positive correlations with SES-CD in patients with CD (*r* = 0.407, *p* = 0.001, Figure 2(D)) and with MES in patients with UC (*r* = 0.489, *p* = 0.001, Figure 2(E)). A paired analysis of serum samples from patients in the NMH group revealed significantly decreased serum reg1α levels after those patients achieved MH [60.7 (40.0–97.0) *vs*. 38.2 (35.1–82.3), *p* = 0.012, Figure 2(F)].

### Ability of reg1α to distinguish between MH and NMH

**Figure 3.**
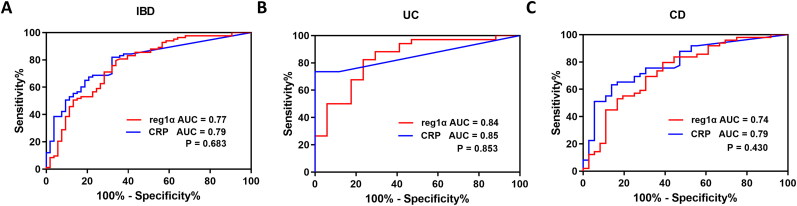
Receiver operator characteristic curves to assess the diagnostic value of reg1α and CRP for endoscopic activity in IBD patients (a), and subgroup of UC (B) and CD patients (C). Comparison the AUCs of reg1α and CRP demonstrated no statistical difference among all patients (*p* = 0.683), UC patients (*p* = 0.853), or CD patients (*p* = 0.430). Abbreviations: AUC, Area under roc curve; reg1α, regenerating islet-derived 1 alpha; CRP, C-reaction protein; IBD, Inflammatory bowel disease; CD, Crohn’s disease; UC, Ulcerative colitis.

ROC analyses were conducted to investigate the ability of reg1α in discriminating MH from NMH. In IBD patients (Figure 3(A)), reg1α demonstrated comparable AUC value to CRP [0.77 (95% CI, 0.68–0.85) *vs.* 0.79 (95% CI, 0.71–0.87), *p* = 0.683, Table 2]. With a cutoff value of 64.40 ng/mL, reg1α exhibited 79.5% sensitivity, 66.0% specificity, 74.3% accuracy, 2.3 PLR and 0.3 NLR. The sensitivity and accuracy of reg1α were significantly higher than those of CRP (cutoff value: 3.0 mg/L) (79.5% *vs.* 67.5%, *p* = 0.049; 74.3% *vs.* 72.1%, *p* = 0.012, respectively). However, no significant difference was observed in specificity between the two biomarkers (66.0% *vs.* 79.3%, *p* = 0.167).

**Table 2.** Discriminatory ability of serum reg1α and CRP for endoscopic activity.

	Cut-off	AUC (95% CI)	Se% (95% CI)	Sp% (95% CI)	Acc% (95% CI)	PLR (95% CI)	NLR (95% CI)
**IBD patients**							
reg1α (ng/ml)	64.40	0.77 (0.68–0.85)	79.5 (69.2–87.6)	66.0 (51.7–78.5)	74.3 (67.0–81.6)	2.3 (1.6–3.5)	0.3 (0.2–0.5)
CRP (mg/L)	3.00	0.79 (0.71–0.87)	67.5 (56.3–77.4)	79.3 (65.9–89.2)	72.1 (64.6–79.6)	3.3 (1.9–5.6)	0.4 (0.3–0.6)
*P*	–	0.683	**0.049**	0.167	**0.012**	**-**	**-**
**UC patients**							
reg1α (ng/ml)	64.65	0.84 (0.71–0.93)	82.3 (65.5–93.2)	76.5 (50.1–93.2)	80.4 (69.5–91.3)	3.5 (1.5–8.4)	0.2 (0.1–0.5)
CRP (mg/L)	3.00	0.85 (0.73–0.94)	55.9 (37.9–72.8)	90.3 (80.5–100.0)	70.6 (58.1–83.1)	5.8 (3.2–10.9)	0.4 (0.3–0.6)
*P*	–	0.853	**0.012**	0.125	**0.001**	**-**	**-**
**CD patients**							
reg1α (ng/ml)	63.97	0.74 (0.63–0.85)	79.6 (65.7–89.8)	61.1 (43.5–76.9)	71.8 (62.2–81.4)	2.1 (1.3–3.2)	0.3 (0.2–0.6)
CRP (mg/L)	3.00	0.79 (0.69–0.89)	75.5 (61.1–86.7)	69.4 (51.9–83.7)	72.9 (63.5–82.3)	2.5 (1.5–4.2)	0.4 (0.2–0.6)
*P*	–	0.430	0.754	0.607	0.424	–	–

Abbreviations: Reg1α, Regenerating islet-derived 1 alpha; CRP, C-reactive protein; IBD, Inflammatory bowel disease; UC, Ulcerative colitis; CD, Cronh’s disease; AUC, Area under the curve; Se, sensitivity; Sp, specificity; Acc, accuracy; PLR, Positive likelihood ratio; NLR negative likelihood ratio; 95%CI, 95% confidence interval; *P*, P value.

In UC subgroup analysis (Figure 3(B)), the AUC of reg1α was comparable to that of CRP (0.84 *vs.* 0.85, *p* = 0.853). With an optimal cutoff value of 64.65 ng/mL, reg1α exhibited superior sensitivity (82.4% *vs.* 55.9%, *p* = 0.012) and accuracy (80.4% *vs.* 70.6%, *p* = 0.001) compared to CRP in monitoring endoscopic activity, while maintaining comparable specificity (76.5% *vs.* 100.0%, *p* = 0.125). The PLR and NLR for reg1α were 3.5 and 0.3, respectively. For CD patients (Figure 3(C)), the AUCs of reg1α and CRP were 0.74 and 0.79, respectively, which were not significantly different (*p* = 0.430). At a cutoff value of 63.97 ng/mL, reg1α demonstrated numerically higher sensitivity (79.6% *vs.* 75.5%, *p* = 0.754) but lower specificity (61.1% *vs.* 69.4%, *p* = 0.607) and accuracy (71.8% *vs.* 72.9%, *p* = 0.424) compared to CRP. The PLR and NLR for reg1α were 2.1 and 0.2, respectively.

### Evaluation of combined index and multivariate model for monitoring endoscopic activity

Our study further investigated the monitoring efficacy of both a combined index (reg1α + CRP) and a ­comprehensive model (incorporating clinical indices, reg1α, and CRP) for monitoring endoscopic activity. In the overall IBD cohort, both combined index and the ­comprehensive model demonstrated significant improvements in sensitivity and accuracy for monitoring NMH compared to CRP alone (all *p* < 0.05; Supplementary Table 1). This pattern remained consistent in the UC subgroup analysis. Notably, in CD patients, the combined index exhibited differential performance characteristics: while specificity significantly improved (91.7% *vs.* 69.4%, *p* = 0.022), this enhancement came at the expense of reduced sensitivity (55.1% *vs.* 75.5%, *p* = 0.001) compared to CRP alone.

### Discriminatory ability of reg1α for endoscopic activity in patients with non- or mild clinical manifestations

**Figure 4.**
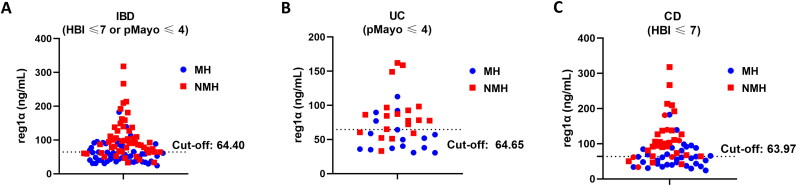
Discriminatory ability of reg1α for endoscopic activity in patients with mild or no clinical manifestations. Reg1α with could correctly distinguish 72.3% (73/101) IBD patients with NMH with a cutoff value of 64.40 ng/ml (a), 74.3% (26/35) UC patients with NMH with a cutoff value of 64.65 ng/ml (B), and 72.7 (48/66) CD patients with NMH with a cutoff value of 63.97 ng/ml (C). Abbreviations: reg1α, regenerating islet-derived 1 alpha; NMH, Non-mucosal healing; IBD, Inflammatory bowel disease; UC, Ulcerative colitis; CD, Cronh’s disease; pMayo score, partial Mayo score; HBI, Harvey-Bradshaw index.

To further verify the clinical value of reg1α as a novel biomarker, we assess the monitoring efficacy of reg1α for endoscopic activity in the subgroup of patients with non- or mild clinical manifestations (defined as HBI ≤ 7 for CD and pMayo ≤ 4 for UC). As illustrated in Figure 4, reg1α has a favourable monitoring ability for NMH in this patient population. At the optimal cut-off value of 64.40 ng/mL, reg1α exhibited superior sensitivity (76.5% *vs.* 56.9%, *p* = 0.031) and accuracy (72.3% *vs.* 68.3%, *p* = 0.012) compared to CRP in monitoring endoscopic activity among IBD patients (Table 3). Although specificity was numerically lower for Reg1α (68.0% *vs.* 80.0%, *p* = 0.238), this difference did not reach statistical significance. Notably, this diagnostic pattern remained consistent in UC patients. However, in CD subgroup, reg1α (cutoff value: 63.97 ng/mL) only presented a numerically higher sensitivity (81.3% *vs.* 71.8%) and accuracy (72.7% *vs.* 71.2%) and lower specificity (64.7% *vs.* 70.6%) than CRP (all *p* > 0.05).

**Table 3.** Discriminatory ability of serum reg1α and CRP for endoscopic activity in patients with non- or mild clinical manifestations.

	Cut-off	Se % (95%CI)	Sp % (95%CI)	Acc % (95%CI)	PLR (95%CI)	NLR (95%CI)
**IBD (HBI ≤7 or pMayo ≤ 4)**						
reg1α (ng/ml)	64.40	76.5 (62.5–87.2)	68.0 (53.3–80.5)	72.3 (63.6–81.0)	2.4 (1.6–3.7)	0.4 (0.2–0.6)
CRP (mg/L)	3.00	56.9 (42.2–70.7)	80.0 (66.3–90.0)	68.3 (59.2–77.4)	2.8 (1.6–5.2)	0.5 (0.4–0.8)
*P*	**-**	**0.031**	0.238	**0.012**	–	–
**UC (pMayo ≤ 4)**						
reg1α (ng/ml)	64.65	73.7 (48.8–90.9)	75.0 (47.6–92.7)	74.3 (59.8–88.8)	3.0 (1.2–7.2)	0.4 (0.2–0.8)
CRP (mg/L)	3.00	31.6 (12.6–56.6)	89.0 (79.4–97.2)	62.9 (46.9–78.9)	2.9 (0.6–4.7)	0.7 (0.5–0.9)
*P*	**-**	**0.021**	0.125	**0.002**	–	–
**CD (HBI ≤7)**						
reg1α (ng/ml)	63.97	81.3 (63.6–92.8)	64.7 (46.5–80.3)	72.7 (62.0–83.4)	2.3 (1.4–3.7)	0.3 (0.1–0.6)
CRP (mg/L)	3.00	71.9 (53.3–86.3)	70.6 (52.5–84.9)	71.2 (60.3–82.1)	2.4 (1.4–4.3)	0.4 (0.2–0.7)
*P*	**-**	0.453	0.791	0.383	–	–

Abbreviations: Reg1α, Regenerating islet-derived 1 alpha; CRP, C-reactive protein; IBD, inflammatory bowel disease; UC, Ulcerative colitis; CD, Cronh’s disease; Se, sensitivity; Sp, specificity; Acc, accuracy; PLR, Positive likelihood ratio; NLR, negative likelihood ratio; HBI, Harvey–Bradshaw index; pMayo, partial Mayo score; 95%CI, 95% confidence interval; *P*, P value.

### Ability of reg1α for detecting MH in patients with normal CRP level

**Figure 5.**
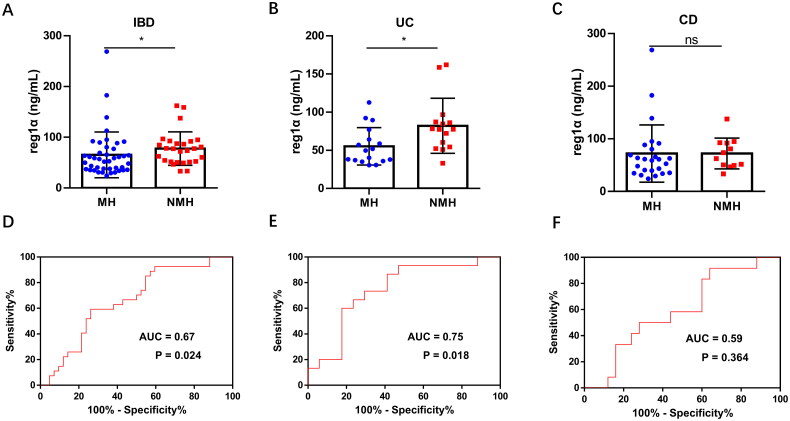
Discriminatory power of reg1α for endoscopic activity in patients with normal CRP levels. (A-C) Comparative analysis of serum reg1α levels between MH and NMH groups: (A) All IBD patients demonstrated significantly elevated reg1α levels in NMH versus MH (*P* = 0.024); (B) UC subgroup showed distinct reg1α elevation in NMH (*P* = 0.018); (C) No significant difference was observed in CD subgroup (P = 0.37). (D-F) In CRP-normal patients, ROC curves and AUCs to assess the diagnostic value of reg1α for endoscopic activity in IBD patients (D), and in subgroup of UC (E) and CD patients (F). *p < 0.05. Abbreviations: ROC, Receiver operator characteristics; AUC, Area under roc curve; ns, not significant; reg1α, regenerating islet-derived 1 alpha; IBD, Inflammatory bowel disease; UC, Ulcerative colitis; CD, Cronh’s disease; MH, Mucosal healing; NMH, Non-mucosal healing.

Previous studies have suggested that CRP levels mediated by IL-6 may remain within normal ranges even during active IBD phases. To address this clinical challenge, we specifically investigated the potential of reg1α as an endoscopic activity biomarker in IBD patients with normal CRP levels (<3.0 mg/L). In 69 IBD patients (42 with MH and 27 with NMH) with normal CRP levels in our study, serum reg1α levels were significantly higher in the NMH group than in the MH group [77.4 (51.7–91.9) *vs*. 54.9 (36.8–78.0), *p* = 0.024, Figure 5(A)]. This differential expression was particularly pronounced in UC patients [77.6 (54.4–87.3) *vs*. 49.8 (36.5–70.7), *p* = 0.018, Figure 5(B)], whereas CD patients demonstrated non-significant numerical differences [68.0 (49.6–92.4) *vs*. 61.0 (37.6–84.4), *p* = 0.378, Figure 5(C)]. The AUC values of reg1α in IBD, UC and CD were 0.67, 0.75 and 0.59, respectively (Figure 5(D–F)). With an optimal cutoff value of 64.40 ng/mL, reg1α demonstrated moderate discriminative capacity for endoscopic activity in CRP-normal IBD patients (sensitivity 59.3%, specificity 69.0%, accuracy 65.2%; Table 4. In 32 CRP-normal UC patients, a cutoff value of 64.65 ng/mL was identified as the threshold for the predictive ability of reg1α for endoscopic activity, with a sensitivity of 66.7%, specificity of 76.5% and accuracy of 71.9%. In 37 CRP-normal CD patients, reg1α (cutoff value: 64.65 ng/mL) showed inadequate efficacy in monitoring NMH with a sensitivity of 50.0%, specificity of 64.0% and accuracy of 59.5%. Notably, the efficacy of reg1α showed no significant difference from clinical indices in either UC or CD patients (both *p* > 0.05).

**Table 4.** Discriminatory ability of serum reg1α and clinical indices for endoscopic activity in patients with normal CRP.

	Cut-off	AUC (95%CI)	Se% (95%CI)	Sp% (95%CI)	Acc% (95%CI)
**IBD**					
reg1α (ng/ml)	64.40	0.67 (0.53–0.79)	59.3 (38.8–77.6)	69.1 (52.9–82.4)	65.2 (54.0–76.4)
**UC**					
reg1α (ng/ml)	64.65	0.75 (0.57–0.92)	66.7 (38.4–88.2)	76.5 (50.1–93.2)	71.9 (54.0 − 76.4)
pMayo	2	0.81 (0.63–0.93)	86.7 (59.5–98.3)	70.6 (44.0–89.7)	78.1 (63.8 − 92.4)
*P*	–	0.602	0.219	0.688	0.140
**CD**					
reg1α (ng/ml)	63.97	0.59 (0.40–0.78)	50.0 (21.1–78.9)	64.0 (42.5–82.0)	59.5 (43.7 − 75.3)
HBI	5	0.71 (0.53–0.84)	33.3 (9.9–65.1)	88.0 (68.8–97.5)	51.4 (35.3 − 67.5)
*P*	–	0.460	1.000	0.549	0.664

Abbreviations: Reg1α, Regenerating islet-derived 1 alpha; CRP, C-reactive protein; IBD, inflammatory bowel disease; UC, ulcerative colitis; AUC, Area under the curve; Se, sensitivity; Sp, specificity; Acc, accuracy; HBI, Harvey–Bradshaw index; pMayo, partial Mayo score; 95%CI, 95% confidence interval; *P*, P value.

## Discussion

This study demonstrated that reg1α correlated significantly with endoscopic mucosal activity. Reg1α has significantly higher sensitivity and accuracy and equivalent specificity for endoscopic activity compared with CRP, especially in UC phenotype. In the subgroup of patients with non- and mild clinical manifestations and patients with normal CRP levels, reg1α can be used as a novel surrogate biomarker of endoscopic activity. To the best of our knowledge, this is the first study to explore the potential clinical utility of reg1α in monitoring MH in patients with IBD.

Recently, MH was proposed as the primary therapeutic goal for IBD because it is associated with a favorable prognosis [5]. However, the evaluation of MH relies mainly on repeated and invasive endoscopic examination. To overcome this challenge, surrogate serum biomarkers, such as CRP, have been widely explored and used in clinical practice because they are cheap, simple to operate, noninvasive, and reproducible. Several guidelines recommend CRP as a serum marker to monitor mucosal inflammation in patients with UC and CD [28,29]. However, the efficacy of CRP as a biomarker of systemic inflammation for monitoring MH is not always satisfactory. A recent study by Mosli et al. demonstrated that the sensitivity of CRP in diagnosing mucosal inflammation in patients with IBD was only 0.49 [15]. Another recent prospective study indicated that CRP is of limited value in monitoring disease activity and could not distinguish between patients with UC with endoscopic remission and those without remission after 5 years of follow-up [30]. Therefore, there is an urgent need to develop more convenient and efficient biomarkers to monitor MH during repeat treatment in patients with IBD.

Reg1α is expressed by pancreatic acinar cells, enterochromaffin-like cells, and all types of gastrointestinal epithelial cells [21,30]. And its receptor exostose-like gene 3 is widely found in the gastrointestinal epithelium [21]. Previous studies reported a significantly high expression of reg1α mRNA in patients with IBD, presenting as the top 10 and 5 differentially expressed genes in CD [31] and UC [32], respectively. The whole-genome transcriptional analysis of colonic biopsies of patients showed that reg1α protein in terminal ileum tissue of patients with UC with endoscopic activity was higher than that in patients with endoscopic remission [32], suggesting that reg1α is closely related to IBD endoscopic activity. In addition, reg1α was exhibited significantly increased expression in UC active involved samples by immunohistochemistry [20]. However, the above findings on the relationship between reg1α levels and IBD activity were mainly based on the results of transcriptional data and immunohistochemistry. In our study, we found that serum reg1α levels in patients with NMH were significantly higher than those in patients with MH for both the CD and UC phenotypes. In addition, serum reg1α levels correlated significantly with endoscopic inflammatory activity scores [MES in patients with UC (*r* = 0.489, *p* = 0.001) and SES-CD in patients with CD (*r* = 0.407, *p* = 0.001), both *p* < 0.001], suggesting that serum reg1α may be a potential serum biomarker for endoscopic activity in patients with IBD.

Previous studies have indicated that elevated CRP levels are highly specific for endoscopic activity; however, its sensitivity is low, especially in UC patients [15,33]. Consistently, the sensitivity of CRP for monitoring endoscopic activity was only 67.5% in patients with IBD. It has been reported that there is remarkable heterogeneity in CRP response; approximately 15% of healthy individuals fail to mount a CRP response [32], which may partly explain the low sensitivity of CRP for monitoring endoscopic activity. Moreover, compared to the strong CRP response in CD, a lower CRP response was detected in UC despite active inflammation [16]. In our study, the sensitivity of CRP level for NMH was much lower in UC group than that in the CD group (55.9% *vs.* 75.5%). The above finding suggests the more urgent need for alternative indicators in UC phenotype. In our study, reg1α showed satisfactory efficacies to compensates for the deficiency of CRP in UC by displaying significantly higher sensitivity and accuracy in distinguishing NMH (82.4% *vs.* 55.9%, *p* = 0.012; 80.4% *vs.* 70.6%, *p* = 0.001, respectively). Additionally, the sensitivity of reg1α for NMH was numerically higher in UC than in CD (82.4% *vs.* 79.6%). CRP, reflecting systemic inflammation, mainly synthesized in the liver *via* circulating IL-6 [34]. CD characterized by transmural mucosal inflammation, tends to elevate circulating IL-6 level to induce a stronger CRP response, whereas UC’s superficial inflammation may largely explain the low sensitivity of CRP for NMH in UC patients. In contrast, REG1α synthesis in the digestive system is induced not only by IL-6 but also by IL-22 and IL-8 [18,21,22], especially IL-22. It has been reported that IL-22 overexpression in UC mucosal inflammatory cells enhances reg1α expression by ­activating its gene promoter, which provided a solid theoretical basis for reg1α to be a novel biomarker of intestinal inflammation. The increased production sites and more stimulating factors may also largely explain our findings that serum reg1α was more sensitive to endoscopic activity than CRP, especially in patients with UC. The EHI, a 13-protein biomarker panel, was developed for monitoring mucosal inflammation, with diagnostic accuracy (AUC) comparable to CRP in both CD and UC patients [35,36]. Similarly, our study found no significant difference in AUC between reg1α and CRP for detecting mucosal inflammation. As a single biomarker, REG1α may offer advantages over the multi-protein EHI panel in cost-effectiveness and methodological stability for clinical implementation.

IBD activity can be evaluated by several levels, including clinical, biochemical, endoscopy and histology. However, patients with clinical remission does not imply endoscopic remission. As endoscopy is an invasive, time-consuming and uneconomical examination, patients with IBD with mild clinical symptoms may refuse to follow-up endoscopic visits, which increases the risk of inflammation progression. Previous studies suggested that CRP failed to differentiate between absent and mild endoscopic activity [15,37]. Several studies have also demonstrated that CRP was only effective in monitoring endoscopic activity in extensive UC but not in proctitis or left-sided UC [38,39], suggesting that the efficacy of CRP may be insufficient in identifying endoscopic activity in patients with non- or mild clinical symptoms. In our study, the sensitivity of CRP was only 56.9% for NMH in patients with non- or mild clinical symptoms in total IBD patients, even extremely low to 31.6% in UC. However, compared with CRP, reg1α could differentiate NMH with significantly higher sensitivity and accuracy (56.9% *vs*. 76.5%, 68.3% *vs*. 72.3%, both *p* < 0.05), especially in the UC phenotype, which reinforced our findings that reg1α can served as a more sensitive biomarker for mucosal inflammation, even for patients with mild clinical symptoms. In clinical practice, elevated reg1α can serve as an early warning indicator, prompting physicians to consider the presence of the potential mucosal inflammation in patients with mild clinical symptoms. It facilitates the implementation of appropriate and timely therapeutic interventions, thereby mitigating the risk of long-term complications, including strictures, perforation, and colorectal carcinoma.

CRP is a typical biomarker of inflammation in patients with IBD. Patients with normal CRP levels are usually considered stable and thus often have delayed or postponed endoscopic evaluation in clinical practice [40,41]. However, a recent study suggested that normal CRP levels may underestimate disease activity in certain patients with CD and common CRP genetic variants (rs2794520 and rs1800947) could limit CRP elevations in active CD [42]. More than 21.9% of paediatric patients with IBD have normal CRP levels, which is more common in UC than in CD [43]. In our study, up to 32.5% (27/83) of IBD patients with NMH presented with normal CRP levels; however, reg1α level still presented a favourable efficacy for MH monitoring in those patients with normal CRP levels, especially in phenotype of UC with 76.5% sensitivity, 71.4% specificity, 71.9% accuracy. The above findings further suggested that reg1α is a promising biomarker of endoscopic activity, even in patients with normal CRP levels.

This study has some limitations. First, it was a cross-sectional study, and changes in serum reg1α levels during the disease course were not determined. However, we believe that our study has some clinical implications in that reg1α is a better surrogate biomarker of endoscopic activity than CRP. However, further prospective studies are required to verify these findings. Second, our study did not assess other biomarkers, such as ESR and faecal calprotectin levels. Third, the sample size of patients with IBD with normal CRP levels was relatively small, which may have introduced a bias in the results. Further research with a larger sample size of patients with IBD with normal CRP levels is needed.

In conclusion, as a cross-sectional study, we demonstrated that reg1α is a reliable and sensitive biomarker of endoscopic activity in patients with UC, even in patients exhibiting non-specific symptoms or normal CRP levels. Further prospective researches with expanded cohort sizes are expected to validate our findings.

## Supplementary Material

Supplemental Material

## Data Availability

The data of this study are available from the corresponding author, Xinying Wang, upon reasonable request.
