# Supplementary material for: Serum regenerative islet-derived protein 1α: a novel and sensitive biomarker for endoscopic disease activity in ulcerative colitis
Source: Ann Med. 2025 Apr 28;57(1):2496404. doi: 10.1080/07853890.2025.2496404 (PMC12039404; doi:10.1080/07853890.2025.2496404)
Supplement: Supplemental Material [file IANN_A_2496404_SM7820.docx]

**Supplement Table 1. Discriminatory ability of the combined index and model for endoscopic activity**

|  | **AUC (95% CI)** | **Se% (95% CI)** | **Sp% (95% CI)** | **Acc% (95% CI)** |
| --- | --- | --- | --- | --- |
| **IBD patients** |  |  |  |  |
| CRP | 0.79 (0.71 - 0.85) | 67.5 (56.3 - 77.4) | 79.3 (65.9 - 89.2) | 72.1 (64.6 - 79.6) |
| Combination | 0.79 (0.71 - 0.86) | 79.5 (69.2 - 87.6) | 66.0 (51.7 - 78.5) | 74.3 (67.0 - 81.6) |
| Model | 0.74 (0.66 - 0.81) | 76.9 (65.3 - 84.6) | 71.7 (57.7 - 83.2) | 74.3 (67.0 - 81.6) |
| *P1* | 0.942 | **0.021** | 0.065 | **0.002** |
| *P2* | 0.203 | **0.045** | 0.344 | **0.027** |
| **UC patients** |  |  |  |  |
| CRP | 0.85 (0.73 - 0.94) | 55.9 (37.9 - 72.8) | 100.0 (80.5 - 100.0) | 70.6 (58.1 - 83.1) |
| Combination | 0.92 (0.81 - 0.98) | 76.5 (58.8 - 89.3) | 94.2 (71.3 - 99.9) | 82.4 (71.9 - 92.9) |
| Model | 0.84 (0.71 - 0.93) | 79.4 (62.1 - 91.3) | 88.2 (63.6 - 98.5) | 82.4 (71.9 - 92.9) |
| *P1* | 0.062 | **0.016** | 0.994 | **0.008** |
| *P2* | 0.811 | **0.008** | 0.500 | **0.002** |
| **CD patients** |  |  |  |  |
| CRP | 0.79 (0.69 - 0.87) | 75.5 (61.1 - 86.7) | 69.4 (51.9 - 83.7) | 72.9 (63.5 - 82.3) |
| Combination | 0.78 (0.68 - 0.86) | 55.1 (40.2 - 69.3) | 91.7 (77.5 - 98.2) | 70.6 (60.9 - 80.3) |
| Model | 0.69 (0.58 - 0.78) | 73.5 (58.9 - 85.1) | 63.9 (46.2 - 79.2) | 69.4 (59.6 - 79.2) |
| *P1* | 0.720 | **0.002** | **0.022** | **0.001** |
| *P2* | 0.06 | 1.000 | 0.727 | 0.238 |

Abbreviations: **IBD**, Inflammatory bowel disease; **UC**, Ulcerative colitis; **CD**, Cronh’s disease; **reg1α**, Regenerating islet-derived 1 alpha; **CRP**, C-reactive protein; **AUC**, Area under the curve; **Se**, sensitivity; **Sp**, specificity; **Acc**, accuracy; **HBI**, Harvey–Bradshaw index; **pMayo**, partial Mayo score; **Combination**, Combined reg1α and CRP indicators (both included as continuous variables in the regression equation); **Model**, Including comprehensive indicators of reg1α, CRP and clinical indices (HBI for patients with CD and pMayo for patients with UC), and at least two of the three indicators are positively judged as active, and the rest are judged as remission; ***P1***, The combination index versus CRP; ***P2***, The Model index versus CRP; **95%CI**, 95% confidence interval; *p < 0.05, **p < 0.001.
